# Needs and Preferences of Partners of Cancer Patients Regarding a Web-Based Psychological Intervention: A Qualitative Study

**DOI:** 10.2196/cancer.4631

**Published:** 2015-12-29

**Authors:** Nadine Köhle, Constance HC Drossaert, Suzan Oosterik, Karlein MG Schreurs, Mariët Hagedoorn, Cornelia F van Uden-Kraan, Irma M Verdonck-de Leeuw, Ernst T Bohlmeijer

**Affiliations:** ^1^University of TwenteDepartment of Psychology, Health & TechnologyEnschedeNetherlands; ^2^Stichting MindfitZwolleNetherlands; ^3^Roessingh Research & DevelopmentEnschedeNetherlands; ^4^University Medical Center GroningenDepartment of Health PsychologyGroningenNetherlands; ^5^VU UniversityDepartment of Clinical, Neuro- & Developmental Psychology, section Clinical PsychologyAmsterdamNetherlands; ^6^VU University Medical CenterDepartment of Otolaryngology/Head and Neck SurgeryAmsterdamNetherlands

**Keywords:** cancer, oncology, partner, needs, web-based interventions, interventions

## Abstract

**Background:**

Evidence-based, easily accessible, supportive interventions for partners of cancer patients are limited, despite the fact that they often suffer from diminished emotional, social, physical, and relational functioning. To develop a new intervention that will fit their demands, it is important to consult potential users.

**Objective:**

To examine partners’ interest in a Web-based psychological intervention and to identify their needs and wishes regarding such an intervention.

**Methods:**

Semistructured interviews were conducted with 16 partners of cancer patients, who varied in terms of age, gender, education, employment, type, and stage of disease. Partners were asked (1) whether they would use a psychological Web-based intervention and which preconditions (maximum time, structure, participate alone or with their partner) it should meet; (2) which functionalities (information, peer support, online psychological counseling) the intervention should contain; and (3) which topics (eg, taking care of oneself) should be addressed. Data were coded by 2 coders independently.

**Results:**

The need for a Web-based intervention varied. Arguments for being interested in a Web-based intervention included the need for acknowledgement; the need for someone they could talk to; and the need for information, tips, and support. Based on their experiences as a partner of a cancer patient, participants would prefer an intervention that is not too time-consuming (about 1-2 hours a week) and which is based on a “step-by-step” approach, meaning that the content of the intervention should match the stage of their partner’s disease. Also, they would prefer a positive approach, which means that the intervention should be a source of hope and energy. Most participants stated that they would prefer to participate without their ill spouse, because they do not want to burden their partners with their own problems. An intervention should contain information and optional peer support. Participants’ opinions about online psychological counseling in the intervention were divided. Arguments for online psychological counseling were that a professional could check on them and they were able to ask questions. Arguments against online counseling were that partners were not in need for guidance or they had enough support from usual care. Topics with the highest priority were “coping with feelings and emotions,” “should I or shouldn’t I spare my partner?,” “communicating with each other,” “asking for help and refusing help,” and “moving on with life after cancer treatment.” Furthermore, participants suggested additional topics of “dare to enjoy” and “acceptance of the patient’s disease.”

**Conclusions:**

A Web-based intervention can be a valuable addition to existing support initiatives for partners of cancer patients. This study provides important information about the content and form of such an intervention. Flexibility and a positive approach seem to be the most important features.

## Introduction

### Partners of Cancer Patients

Cancer not only affects the patients’ lives, but also the lives of their loved ones. Partners of cancer patients may suffer from diminished emotional, social, physical, and relational functioning [1-11]. The couples’ relationship often changes because of shifting roles and responsibilities [3,4], feelings of inequality [5,12], reduced social activities, less financial resources [6,7], and a decrease of sexuality and intimacy [8]. Problems often occur when patients and partners avoid talking about the disease, their feelings, and changes in their relationship [9]. Recent studies have shown that clinical levels of psychological distress are highly prevalent in partners of cancer patients (especially in female partners) and can even be higher than the levels experienced by patients themselves [1,2,13]. Cancer can directly and indirectly affect the physical well-being of partners [6], because many partners have barely time to relax and they often neglect their own health [9].

Despite the known multiple and serious effects of cancer on partners’ lives, the availability of evidence-based, easily accessible, supportive interventions for partners of cancer patients is still limited. The interventions that do exist vary widely in their scope, aims, target groups, intensity, used methods, and theoretical frameworks [9,14,10]. Northouse et al [9] classified the interventions into 3 major types: psychoeducation, skills training, and therapeutic counseling. The majority of the interventions belong to the first type, and these primarily strive to provide information about the optimal patient care. Skills training tries to improve skills regarding coping with the situation, communication, and problem solving. Therapeutic counseling, finally, aims to address concerns regarding cancer or caregiving. The interventions also vary widely in terms of how demanding they are: most interventions are delivered as face-to-face visits, with the majority provided in a clinical setting, they take between 1.7-18 hours; they comprise between 2-16 sessions; and they last for 1.2-56 weeks from first to last session [9]. Most existing interventions are developed for couples (both partners and cancer patients) and since usually no differentiation is made between their needs, the focus is inevitably often on the patients’ care and well-being. Only a few interventions have primarily addressed partners’ well-being [9,14]. Furthermore, partners of cancer patients often make no or only limited use of existing interventions [14]. Many of the interventions described in the meta-analysis of Northouse et al [9] and reviews of Ussher et al [14] and Applebaum and Breitbart [10] report difficulties with inclusion or high dropout rates. Reported reasons for low participation are, for example, that partners are often not aware of their own health complaints and that they therefore do not feel in need of support [15]. Participation is also connected to the demands of the illness, when the demands are high (eg, intensive treatment), existing interventions seem to ask too much from the partners and they will not participate [16]. Other identified barriers to make use of the offered resources are being unaware of existing sources, being reluctant to ask for help or to talk about sensitive topics, and being afraid that their own requests may affect the care of the patient [17]. Another possible explanation may be that the existing interventions do not fit to the specific needs of partners of cancer patients [14]. Ussher et al [14] recommend prior needs assessments before development.

Another recommendation was to examine the potential for using the Internet to deliver interventions to the caregivers of cancer patients [9,10]. The Internet offers new opportunities to deliver easily accessible and (cost-) effective supportive interventions. Possible advantages of Web-based interventions include a low threshold, flexibility, and possibilities to follow the intervention at any time that suits the client [18]. These features might be especially important for partners of cancer patients since they have less time for their own mental and physical health. The Internet also bears the possibility to tailor information and feedback to the individual needs of a client. This may be beneficial to partners of cancer patients because they are only confronted with information that is relevant to them [19]. Despite these benefits, the availability of Web-based interventions for partners of cancer patients is also still limited [20]. To the best of our knowledge, no studies exist that have examined the views and opinions of partners regarding a Web-based intervention.

### Aim of the Study

Accordingly, the aim of this study was to examine partners’ interest in a Web-based psychological intervention and to identify their wishes, desires, and needs regarding such an intervention. This study focused on the following questions: (1) “Is there a need for a Web-based intervention and which preconditions (maximum time, structure, participate alone or with their partner) should it meet?”; (2) “Which functionalities (information, peer support, psychological guidance) should the intervention contain?”; and (3) “Which topics (eg, taking care of oneself) should be addressed?”

## Methods

### Study Design and Ethical Approval

A qualitative research design was chosen to gain insights into the wishes, desires, and needs of partners of cancer patients regarding a Web-based psychological intervention. Semistructured interviews were conducted. The Ethics Committee of the University of Twente (Behavioural, Management, and Social Sciences) provided ethical approval for this interview study and the study was conducted according to the declaration of Helsinki.

### Participants and Procedures

Partners of cancer patients were recruited in a large hospital in the region of Twente, an area in the east of the Netherlands. A nurse practitioner informed partners of cancer patients of the ongoing study and she handed out information leaflets. In case partners were interested in participating, they had to fill out a reply card with their name and telephone number on it, and return it to the nurse practitioner. Subsequently, the nurse practitioner contacted the researchers so that they could get in touch with the partner. Additionally, partners were recruited through convenience sampling. Partners were people from the network of the researchers and they were called and asked if they wanted to participate in this study. In case they were interested, they received an information leaflet by mail or email and after reading the information they could decide if they still wanted to participate. Once the participants had given their informed consent, they were interviewed. The interviews took place at the participants’ homes. There were 2 researchers (NK and SO) that conducted 16 interviews together. Both researchers are psychologists and were trained in conducting interviews. Initially, the researchers proposed to interview the partner alone, without their ill spouse. However, during 3 interviews the (patient) partner was also present, because the partner explicitly wanted the patient to be there. After the 16 interviews data saturation was reached, meaning that no more new information was found [21]. All interviews were audio-recorded—with the prior permission of the participants—and the audiotapes were transcribed verbatim.

### Interview Scheme and Mock-Ups

**Figure 1:**
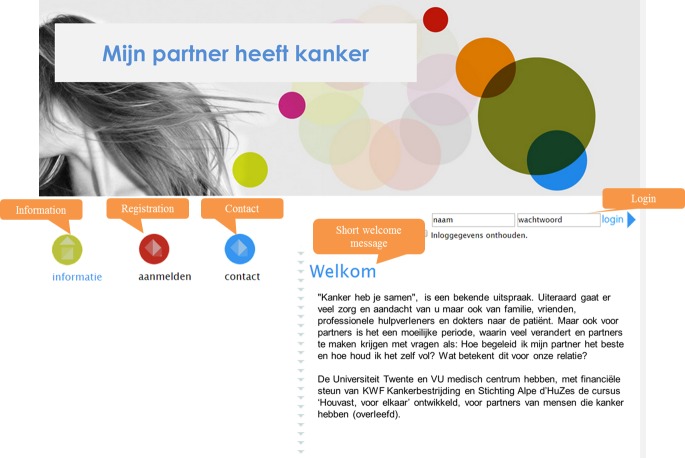
Mock-up of a possible Web-based intervention.

**Figure 2:**
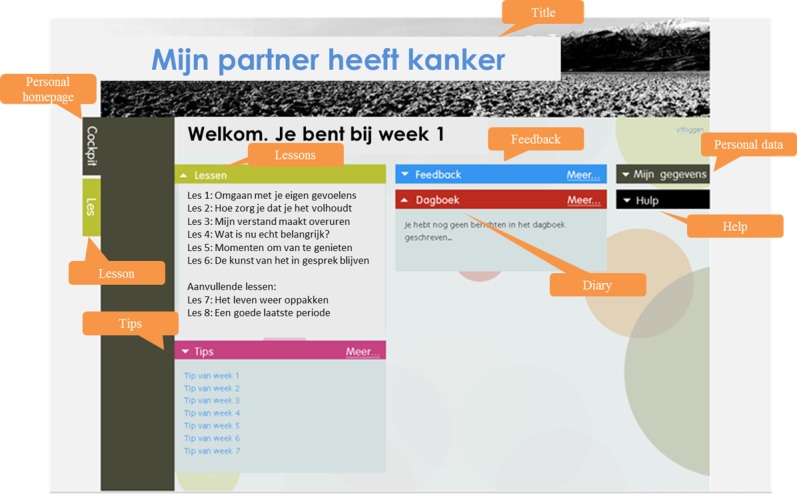
Mock-up of a personal home page (after participants have logged in).

All interviews started by asking participants to introduce themselves and to give a short overview of their partner’s disease and how this had affected them personally. After that, partners were asked about their ideas and opinions about a Web-based psychological intervention. As many participants had difficulties conceptualizing the idea of a Web-based intervention, 2 mock-ups of a possible Web-based intervention for partners of cancer patients were shown to the respondents. These mock-ups were based on an existing Web-based intervention called “Living to the full” (Figures 1 and 2 show this) [22-24]. Participants were encouraged to elaborate on their motives for (not) wanting a Web-based intervention. With an open-ended question, we asked the participants which functionalities a Web-based intervention should contain. We continued by asking their opinion about the preselected functionalities: information, peer support, and online psychological counseling. Regarding the preconditions of the intervention, we invited participants to reflect on the following issues: maximum time, structure, and participate alone or with ill partner. Participants were encouraged to motivate their answers and to add other functionalities or preconditions. Finally, we asked partners which topics should be addressed in a Web-based intervention. First, an open question was posed. In addition, the researchers had prepared 9 cards with words of potential topics. These topics were based on literature and suggestions of 5 experts in the field who we have consulted beforehand. The topics were: (1) coping with feelings and emotions; (2) taking care of oneself; (3) sparing your partner or not?; (4) communicating with each other; (5) sexuality and intimacy; (6) asking for help and refusing help; (7) moving on with life after cancer treatment; (8) living with cancer; and (9) if the end is near. Participants were asked to pick those cards which were possibly relevant to them and which should be targeted in a Web-based intervention. Participants were asked to motivate their choice. Also, they were encouraged to add more topics with an extra “empty” card. At the end of the interview, participants completed a short questionnaire about sociodemographics (such as gender, age, education, employment). The interviews took between 40 minutes and 2 hours, with an average duration of 65 minutes.

### Data Analysis

There were 2 coders (NK and SO) that independently coded all transcripts. First, the coders read and reread all transcripts to familiarize themselves with the content. Then, relevant fragments were selected and coded into one of the 4 main themes: (1) need for Web-based intervention; (2) preconditions; (3) functionalities; and (4) topics. Subsequently, all fragments were further categorized into subthemes using inductive analysis. Inductive analysis means that the subthemes derive from the data, instead of from predefined categories. After every 5 transcripts, the coders met to discuss their categories. When coders disagreed about the categorization, discussion took place until consensus was reached. The final categories were defined on the basis of consensus between the 2 researchers.

## Results

### Participants

The characteristics of the 16 participants and their ill partners are listed in Tables 1 and 2. Participants were heterogeneous regarding gender, age, education, and employment. The partners of the participants were diagnosed with a variety of cancers, they varied in prognosis, and most of them were not under treatment (2 under surveillance, 6 in recovery) when the interviews took place. There were 3 of the participants that were widow/widower and used recollection to answer our questions.

**Table 1:** Characteristics of the participants (N=16).

Characteristics		N	%^a^
**Gender**			
	Male	10	63
	Female	6	38
**Age**			
	Mean (SD)	51.7 (12.8)	
	Range in years	30-68	
**Religious**			
	No	2	13
	Yes	13	81
	Unknown	1	6
**Children**			
	No	2	13
	Yes	14	88
**Education**			
	Low	7	44
	Medium	4	25
	High	5	31
**Employment**			
	Full- or part-time work	10	63
	Retired	3	19
	Disabled	0	0
	Other	3	19

^a^Percentages may not total 100 due to rounding

**Table 2:** Characteristics of the ill partners (the patients) (N=16).

Characteristics		n	%
**Age**			
	Mean (SD)	52.5 (13.5)	
	Range in years	32-71	
**Type of cancer**			
	Lung cancer	1	6
	Acute lymphatic leukemia	1	6
	Hodgkin’s lymphoma	1	6
	Prostate cancer	1	6
	Ovarian cancer	1	6
	Testicular cancer	1	6
	Breast cancer	2	13
	Kahler’s disease	1	6
	Cervical cancer	1	6
	Brain tumor	2	13
	Skin cancer	2	13
	Non-Hodgkin’s lymphoma	1	6
	Oesophagus cancer	1	6
**Undergoing treatment**			
	Yes	5	31
	No	8	50
	Deceased	3	19
**Prognosis (self-reported)**			
	Good	6	38
	Poor	4	25
	Uncertain	3	19
	Deceased	3	19

### Need for a Web-Based Psychological Intervention

There were 2 of the participants that gave no answer to the question if they were in need for some kind of a Web-based intervention. One of them had no Internet access at home and the other did not use the Internet. They also had difficulties in imagining what a Web-based intervention would look like, even after being given a short explanation of a possible intervention and after being shown the mock-ups. We decided nevertheless to continue the interview with these partners, because we thought these might still give us valuable information about, for example, which topics should be addressed in a Web-based intervention for partners of cancer patients.

Among the remaining participants (n=14), the need for a Web-based intervention varied. There were 6 of them that explained that they would like some kind of Web-based intervention, 4 had ambivalent feelings toward such an intervention, and 4 partners were not interested. Participants’ arguments for being interested in a Web-based intervention could be divided into 3 categories: (1) the need for acknowledgment; (2) the need for someone you can talk to; and (3) the need for information, tips, and support regarding their specific needs as a partner of a cancer patient, as illustrated in the following citations,

I really missed something offered to me as a partner of a cancer patient.

Female, 63, partner had Oesophagus cancer

Sometimes you need to tell your story. But my friends were all in a different situation, they just became parents or they were pregnant. A totally different life situation. Therefore, they had problems talking to me. And for my part, I didn’t want to be a burden to them either.

Female, 30, partner had skin cancer

I was looking for acknowledgment. Acknowledgment for all the emotions that you experience as a partner of a cancer patient. Fear, anger, helplessness[...]

Female, 51, partner died of acute lymphatic leukemia

Participants who had ambivalent feelings toward a Web-based intervention mentioned various arguments. One of them said that she was not sure whether she had the need for an intervention targeting the partner or not. This need actually changed from moment to moment. However, she was sure that she would prefer face-to-face contact instead of Web-based support. Also, for her it felt wrong to spend some personal time while her partner was ill and she mentioned that she was afraid of losing valuable time with him.

Time was too valuable to participate in a Web-based intervention because we already knew that he wouldn’t get better anymore.

Female, 55, partner died of lung cancer

Another participant said that it was difficult for him to give an answer to this question because—at the time his wife was ill—he was not aware of the fact that he actually needed support. His mere focus lied on his wife’s health and her needs and he wanted to be the “hero” for her. His own (health) problems were not important to him at all. He said that the choice to make use of such an intervention would depend on the way this intervention would have been offered to him, see the following quote,

It is difficult to give an answer to this question, because I think it depends on how such an intervention was offered to me. If it was something like a therapy or help program...? Well look, as partner of a cancer patient you don’t know that you are actually in need for help or, rather, you are convinced that you are not in need for help[...]in my opinion, I tried to be the hero. And it doesn’t fit in the role of a hero to participate in a help program[...]I think “support” is a more appropriate word to use[...]I would have been interested in something that aims to improve my skills as caregiver.

Male, 43, partner died of ovarian cancer

Another participant welcomed the idea of a Web-based intervention for partners of cancer patients, because he was convinced that a lot of partners are in need of such an intervention. However, he was not sure if he also shared that need. In his opinion, he and his wife managed the situation well (they indicated that they had a down to earth approach to cope with the disease), but they were not sure if this way of coping was the most appropriate and effective way. He guessed that he probably would take a look at what such an intervention could offer him. In particular, he would be interested in acknowledgment.

But sometimes I am wondering, in the beginning people sometimes said to us “that you can be so down to earth in coping with it (the disease)”. Then you can ask yourself “who is the crazy one?”. Maybe our approach is not the right one at all.

Male, 30, partner had non-Hodgkin’s lymphoma

A participant explained that she would only be interested in an intervention that targets effective ways of coping with the disease instead of talking about the situation and problems again and again.

If you are there [at a meeting with a psychologist], I’ve heard that you have to talk about your problems every time[...]You always have to tell the same old story and I think it is important to look forward. It is not necessary to look back at what has happened in the past[...]How can you cope with it? How can you process it without constantly talking about the problem again?

Female, 68, partner had skin cancer

There were 4 participants that explained that they were not interested in a Web-based intervention, because they simply were not in need for support. There was a participant, for example, that explained that she is engaged in a variety of social activities (eg, choir, yoga class) and that the situation is not affecting her in a way that she would need help. Furthermore, she trusts the medical staff of her husband and accordingly she never used the Internet for looking up information about her husband’s disease. Other arguments mentioned were that participants think that they were not “the type” to participate in such an intervention, or that they want to spend all their time with their spouse instead of participating in any kind of support. Yet, 2 of the 4 participants were convinced that other partners would be interested in an intervention that targets their specific situation as a partner of a cancer patient. All the arguments regarding the need for a Web-based psychological intervention are listed in Table 3.

**Table 3:** Arguments regarding the need for a Web-based psychological intervention.

Variable	Arguments pro	Arguments con
Need for Web-based intervention	Need for acknowledgment	Experiencing no problems or not being aware of any problems
	Need for someone you can talk to	Having sufficient support from social network or own coping-strategy seems fine
	Need for information, tips, and support	Not wanting to lose valuable time with ill partner or feeling that it is wrong to spend personal time while partner is ill
		Being afraid of too much negativity through rehashing the problem; intervention seems not appealing

### Preconditions of a Web-Based Intervention

There were 4 participants that gave no answer to these questions, because they had no computer at home (n=1), they had no experiences using the Internet (n=1), or they were not able to give an indication (n=2). The majority of the remaining participants (n=10) reported that the intervention should not be too time-consuming. It appeared that partners who are more certain about their need for a Web-based intervention would be willing to spend more time on it. There were 8 participants that mentioned that they could spend about 1-2 hours a week in an intervention,

I think that it is really important, so one and a half hours is not too much. This doesn’t mean that you have to spend the time without a break.

Female 58, partner has brain tumor

There were 2 participants that indicated that they were willing to spend about 3.5 hours a week in such an intervention.

Regarding the structure of the intervention, 3 participants explicitly mentioned that they would prefer a “step-by-step” approach, which means that the content of the intervention should match the stage of their partner’s disease. For example, participants did not want to receive information about the terminal phase if their partner had just been given a diagnosis of cancer, as is illustrated by the following quote,

Try to look at it step-by-step. This is a tip I received from my brother. Try not to think too far ahead and try to avoid the thought “what if...?” and all the bad scenarios. Be aware of the things that are really important at this moment.

Male, 43, partner died of ovarian cancer

Also, participants mentioned that the intervention should have a positive approach. According to them, thinking positively and accentuating what still can be done, instead of what no longer can be done, is a source of hope and energy for both the partner and the cancer patient,

[...]as long it is a little bit positive. I’m not interested in the negative things. Because they only result in a depressed mood.

Female, 68, partner had skin cancer

There were 8 of the participants that preferred to participate in the intervention without their ill spouse. One of the reasons for this preference is that their ill spouse is not in need for help. Another reason is that they did not want to burden their partners with their own problems, and that they could express their feelings and emotions more freely if they participated in the intervention alone.

I would prefer to participate in the intervention on my own. I think this is of added value. I would have the chance to tell my story and show my emotions freely without anyone knowing.

Male, 30, partner had non-Hodgkin’s lymphoma

Furthermore, one partner argued that partners’ and patients’ needs are different and that it is therefore difficult to combine both in one intervention,

No, for my partner it is different. He really has a different point of view, because he is the patient. And he is focused on himself, and as a partner you have to focus not only on yourself but also on your partner. And you have to manage in daily life. I think that these are two different things.

Female, 63, partner had Oesophagus cancer

There were 3 of the participants that felt that it would be important to participate in the intervention together with the ill spouse. They explained that the disease affects the lives of both partners and that it is essential to cope with the situation as a couple.

I think you should do this together, because you are in this situation together.

Female, 58, partner has a brain tumor

There were 4 interviewees that suggested that participants should be able to choose whether they want to participate alone or together with their partner, for example,

I think you should be free in this choice. I have the need to participate in such an intervention, but my partner doesn’t. In this case it is not necessary to participate together.

Female, 30, partner had skin cancer

### Desired Functionalities of a Web-Based Intervention

#### Information

The majority of the participants (n=14) were interested in information (see Table 4). Relevant medical information should come from a reliable source, should be presented in a clear and intelligible way, and it should match their partner’s stage of disease. According to 7 participants, it would be sufficient to include links to other reliable websites (eg, the website of the Dutch Cancer Society). There were 7 participants that doubted if medical information would be actually necessary, because they already received a lot of medical information in the hospital, or because they feared that the presented information would be too general. Alongside the medical information, participants also expressed a need for information and practical tips about what it means to be partner of a cancer patient (this is further described in the section “Important Topics to Be Addressed by the Intervention” and Table 5).

**Table 4:** Arguments and preferences regarding the various functionalities of a Web-based psychological intervention.

Variable		Arguments pro	Arguments con	Preferences
**Functionalities**				
	Information	Being informed about all aspects of disease	Information overload	Medical and practical information is preferred
		Being informed about what it means to be a partner of a cancer patient	Information usually too general	From reliable source
		Practical tips can be helpful		Be clear and intelligible
				Match partner’s stage of disease
				Links to relevant websites are sufficient
	Peer support	Acknowledgment	No time to support others	Possibility to read experiences and tips of other partners
		Confirmation	Problems with managing own problems	Possibility to participate (anonymously) on Web-based platforms
		Support	Doubting helpfulness of peer support	
		Someone who will listen	Afraid of being confronted with negative experiences	
	Online psychological counseling	Signaling	Professionals’ advices in the hospital are sufficient	Feedback tailored to personal situation
		Improving motivation	No further support is needed	Feedback from reliable person
		Possibility to ask questions	Term “psychological guidance” is too heavy	
			No need; satisfied with regular health care	

**Table 5:** Relevant topics for a Web-based intervention, according to the partners (n=16).

Topic	n
Coping with feelings and emotions	16
Should I or shouldn’t I spare my partner?	16
Communicate with each other	16
Asking for help and refusing help	16
Moving on with life after cancer treatment	16
Sexuality and intimacy	13
Taking care of oneself	10
Living with cancer	10
The end is near	10

#### Peer Support

The majority of the participants (n=10) were interested in some form of peer support (see Table 4). They were looking for acknowledgment, confirmation, support, and someone who would listen to them, as expressed by these quotes,

Look for other partners of cancer patients. They will understand you immediately and can help you. You will definitively find acknowledgment.

Female, 51, partner died of acute lymphatic leukemia

The information you receive is valuable, because everyone is looking for confirmation[...]You are doing something instinctively, but you are uncertain if this is the right thing to do. You want to know how other partners handle it.

Female, 63, partner had Oesophagus cancer

Often it is enough that somebody is listening. People often only want to tell their story.

Female, 51, partner died of acute lymphatic leukemia

Opinions about the best form of peer support varied, however. Some indicated that it would be sufficient to read about experiences of partners of cancer patients. Others wanted to actively participate on Web-based platforms (whether anonymously or not), because they wanted to share their experiences with other partners of cancer patients or they appreciated the personal contact for understanding, support, and acknowledgment.

However, a group of participants were not sure about their interest in contact with peers (n=4) or they were not interested in peer support at all (n=2). Arguments against peer support were that it was enough for them to cope with their own situation and that they did not have time to support others.

I don’t know how other partners handle this issue, but I definitely had no time for it[...]I’m not sure how much capacities I had left at that moment to listen to another person’s story. But I guess very little.

Male, 43, partner died of ovarian cancer

In addition, they doubted whether experiences of other partners of cancer patients would be helpful to them, and they were afraid to be confronted with negative experiences, as illustrated with the following quotes.

I have to confess that I tried to avoid peer support, because there were always people with even worse stories. And if you are in a period of hope and the other person is in a period of despair, this can negatively affect your own mood and hope.

Male, 43, partner died of ovarian cancer

I think that peer support about medical issues can be negative. It scares people about situations, which might not have been come up yet.

Male, 30, partner had non-Hodgkin’s lymphoma

#### Online Psychological Counseling

There were 2 participants that gave no answer to this question. Of the remaining participants, opinions about online psychological counseling varied (see Table 4). There were 9 participants that were positive about some kind of online psychological counseling. First, they liked the idea that a professional could check on them and would be able to signal if something went wrong (eg, if their mental health was deteriorating).

I think this is quite important. Imagine that someone is writing something in a depressed tone. Then a psychologist would be able to intervene and check on him or her.

Male, 30, partner had non-Hodgkin’s lymphoma

Second, they thought that a personal online counselor could improve their motivation to complete the Web-based intervention, and third they liked the idea that they would be able to ask questions, as illustrated in the following quotations.

No obligations and flexibility are necessary, but it is also important that there constantly is someone who - how should I call it - someone who wakes you up if necessary.

Male, 43, partner died of ovarian cancer

Yeah, I think that people need this and that they would like the idea to rely on it (the psychological guidance)[...]The website shouldn’t just say: “Deal with it”. It is necessary, well look, if they pick a topic and have a lot of questions about it, then these questions need to be answered.

Female, 58, partner has a brain tumor

However, 3 of the participants also mentioned that they would prefer feedback that is focused on their personal situation. General feedback would not be enough to satisfy their needs. Furthermore, 1 participant mentioned that he would prefer guidance from a person he knows, definitively someone who is capable, and knows how things work. There were 3 interviewees who also mentioned that online psychological counseling should not be mandatory, but offered as a possibility.

There were 5 participants (3 of these were generally not in need of a Web-based intervention) that were not interested in online psychological counseling, because they had no need for it or they were already satisfied with the help given by doctors and nurses in the hospital and they felt they did not need any further support**.**

We encouraged participants to bring up any other functionalities. However, they didn’t come up with anything else.

### Important Topics to Be Addressed by the Intervention

As described earlier, participants were asked to choose topics that were relevant to them and should be addressed in a Web-based intervention. Participants reported that all the proposed topics were valuable to partners of cancer patients. However, they emphasized the importance of the topics “coping with feelings and emotions,” “should I or shouldn’t I spare my partner?,” “communicating with each other,” “asking for help and refusing help,” “moving on with life after cancer treatment,” and “sexuality and intimacy” (see Table 5). Furthermore, 4 participants suggested an additional topic “dare to enjoy”. The topic refers to enjoying those things that they still can do, instead of regretting what they cannot do anymore. This is an important source of hope and energy for the cancer patient as well as for the partner. There was 1 participant that added the topic “acceptance of the patient’s disease”. She had difficulties accepting their partner’s disease and she wished to get some help with that process.

## Discussion

### Need for a Web-Based Intervention

In this study, we examined partners’ interest in a Web-based psychological intervention, and their needs and wishes regarding such an intervention. We found that the need for a Web-based intervention varied. Arguments for being interested in a Web-based intervention were: (1) the need for acknowledgment; (2) the need for someone who would listen; and (3) the need for information, tips, and support. Arguments against such an intervention were: (1) not experiencing any problems or not being aware of any problems; (2) having sufficient support from the social network or their own coping-strategy seems fine; (3) not wanting to lose valuable time with their partner or feeling that it’s wrong to spend some personal time while the partner is ill; and (4) being afraid of too much negativity through rehashing the problem or an intervention seems not appealing. These results correspond with findings of previous research among cancer caregivers. For example, Harding and Higginson [25], Ussher et al [14], and Northouse et al [9] have found that many informal cancer caregivers are not asking for help, because they are often not aware of their own needs and problems, and they are mainly focused on the well-being of the patient. We think that it is of the utmost importance that we create more awareness for the challenging situation partners (or other caregivers) of cancer patients are confronted with every day. Both partners and the general public should be alerted (eg, through awareness campaigns) about the effects and consequences that often come along with a diagnosis of cancer. Also, partners should be informed about the different possibilities to receive help (eg, social workers, psychologists, nurse practitioners, Web-based interventions), as some partners in our study explicitly stated that they were not aware of any initiatives. By offering (information about) different kinds of support, we can ensure that everyone receives that kind of support that he or she needs and prefers. For some cancer caregivers, it is probably enough to be acknowledged that cancer may also affect their lives. Others may wish to consult a psychologist or they have a good relationship with their general practitioner, medical staff, or they receive sufficient support from their network. We think that a Web-based intervention can help caregivers who have little time to seek help; who experience a high threshold to consult a psychologist; who want to stay anonymous; or who want to check if they are in need for support before actually seeking help from a health care professional.

In our sample, we have seen that most of the partners had no or only little experience with e-Health interventions and also there were misconceptions about psychological interventions in general (eg, the idea that psychologists only want to rehash the problem). To inform partners about the possibilities of a Web-based intervention and to overcome misconceptions, we would recommend the use of both written and visual (eg, demonstration video) information about the content and nature of such an intervention.

We can conclude that partners of cancer patients differ in their opinions about the need for a Web-based (or any other) psychological intervention. Our data suggest that more awareness for the situation of cancer patients is needed, and information about existing options for support is lacking. In addition, our data show that there is a considerable group of partners who would be interested in a Web-based psychological intervention.

### Preconditions

Overall, participants reported that an intervention should not be too time-consuming. They were afraid of losing valuable time with their partners and they also emphasized that they were already challenged with managing caregiving responsibilities and everyday tasks. According to the participants, they were able to spend about 1 to 2 hours a week on a Web-based intervention. For the successful implementation of such an intervention, it is important to meet the specific needs of the partners. The advantages of Web-based interventions (low threshold, high accessibility, flexibility) will be useful to fulfill these needs.

As far as the content of the intervention is concerned, the participants in our study would prefer a step-by-step approach. This means that the content should match the patient’s stage of disease. The participants would also prefer a positive approach. They explained that they are confronted with enough misery (almost) every day and that it would be important that a Web-based intervention would also focus on positive things in life and in their specific situation. They indicated that such an intervention should be a source of hope and energy. This preference fits in with the developments in the field of psychology. Psychology traditionally focused on dysfunction. Positive psychology, in contrast, aims to focus on the positive features that make life worth living such as hope, optimism, happiness, and well-being [26]. Accordingly, we think that it could be of great value if an intervention for partners of cancer patients is based on concepts stemming from positive psychology, such as acceptance, values, resilience, mindfulness, and self-compassion.

As described earlier, most available supportive interventions aim at the couple (patient and partner) and usually no differentiation is made between their needs [9,14]. However, we have found that most of our participants would prefer to participate alone. They doubted that patients’ and partners’ needs could be combined in a single intervention. A small group of participants would prefer to participate together with their ill spouse because the disease affects both their lives. These participants explained that it is essential to cope with the situation together. According to these different preferences, we would recommend a flexible approach (participating alone versus participating together) for a future Web-based intervention for partners of cancer patients.

### Desired Functionalities

Participants in our study indicated that a Web-based psychological intervention should contain information as well as peer support. We found that participants were mainly interested in information and practical tips about all aspects of the disease and the consequences of being a partner of a cancer patient, coming from a reliable source. Previous research among partners of cancer patients has shown similar findings [27,28]. However, some partners in our study doubted if medical information is necessary for a Web-based intervention. They indicated that they have already received a lot of information in the hospital, or they feared that the information would be too general. Other researchers reported a similarly wide range of information needs of partners of cancer patients [7,29,30]. The different preferences regarding information needs should be considered in a Web-based intervention for partners.

Most participants were interested in peer support because they were looking for acknowledgment, confirmation, support, and someone who would listen. However, their wishes regarding the type of peer support varied. Whereas some participants would prefer the possibility to merely read experiences and tips of other peers and to stay anonymous, other participants preferred to actively participate in Web-based platforms. Rozmovits and Ziebland [30] also showed the general need for peer support in a study on the information needs of cancer patients. In this study, participants reported that having access to the experiences of peers was generally positively valued because it results in reduced feelings of fear and isolation during their illness, and it was both informative and reassuring. Furthermore, van Uden-Kraan et al [31] found that active participation in a Web-based support group by sending postings and nonactive participation by mere reading of postings from others are equally effective.

Despite the positive effects of peer support, some partners of our study indicated they had no interest in contact with other peers. They explained that they struggle with their own situation and that they did not have time to support others. Besides, they doubted whether the experiences of other partners would be helpful to them. These results are in line with various previous studies [32,33]. It seems that partners have ambivalent feelings toward peer contact: they do feel the need, yet they are afraid of being confronted with negative stories of other peers. Therefore, we would advocate that a future Web-based intervention for partners of cancer patients should offer the possibility to get in touch with peers. However, we would recommend a flexible approach in participation where partners will be able to engage in the type of contact with peers that actually matches their wishes (participation vs nonparticipation; active vs passive peer support) and type of peer support (eg, Web-based platform vs private messages).

The need for online psychological counseling during participation in a Web-based intervention varied. Most of our participants liked the idea that a professional would guide them through the intervention, but others rate the presence of a professional as unnecessary. We can conclude that there are different preferences regarding psychological guidance. Recent studies have revealed that personal guidance is essential for the effectiveness of, and adherence to, eHealth interventions [34-37]. Yet, there is no consensus about the amount or form of support. For example, a study on the self-help intervention “Living to the full” with email support has indicated that short support messages were as effective as more extensive counseling [38], and a study of Kelders [39] has shown that automated support (consisting of a weekly feedback message) was as effective as a weekly feedback message given by a personal online counselor. However, more research in this field is needed to, for example, examine whether personal guidance is more effective for certain groups of partners. For a Web-based intervention for partners of cancer patients, it would definitely be useful if the different preferences regarding online psychological counseling could be considered.

### Topics

Our participants agreed about the relevance of all the mentioned topics. They were especially interested in topics like “coping with feelings and emotions,” “should I or shouldn’t I spare my partner?,” “communicating with each other,” “asking for help and refusing help,” and “moving on with life after cancer treatment”. Furthermore, participants suggested extra topics of “dare to enjoy” and “acceptance of the disease”.

In line with the fact that partners are (often) unaware of their own health complaints and therefore do not ask for help [9,14,40], participants in this study rated the topic “taking care of oneself” as less important than the other topics. Based on these outcomes, we think it is essential that an intervention targeting this group should be framed as informal and easily accessible support, from a positive perspective.

### Limitations

There are some limitations to this study. First of all, this qualitative study was performed with a rather small number of respondents. We aimed to explore the needs and wishes of a group as heterogeneous as possible. We feel that we have succeeded in this effort as a wide range of people (in terms of gender, age, type, stage of disease, treatment) participated. However, the selective group of participants may not be representative for all partners of cancer patients. Therefore, it may be worthwhile to develop a quantitative questionnaire based upon the outcomes of this study, to corroborate the results in a larger sample of partners of cancer patients. In a quantitative study, it would also be possible to identify variables (eg, gender, age, type, stage of disease, treatment) that are related to the intention to make use of a Web-based intervention.

Second, during recruitment, partners were told (in the information leaflet) that the interview was about a Web-based intervention. This could have led to selection bias. It might have been that partners of cancer patients who were not (regularly) using the Internet would have been less likely to participate.

Third, it should be noted that during 3 interviews the patient was also present. We agreed to this when the partner wanted their spouse to be present. However, it could have been possible that the presence of the patient had influenced the partner’s answers. Perhaps they were more cautious talking about their personal needs and wishes in order to protect their partner’s feelings.

Fourth, we have to note that 3 of our participants were widow/widower and that they used recollection to answer our questions, whereas the other participants used their current state. We asked the 3 partners to report on what would have been helpful to them in case their partner was still alive. We do not know for sure if these answers would have been the same when their partners were still alive, but it appears from our study results that the opinions of these 3 participants are in line with those of the other participants.

At last, it might have been difficult for partners to decide upon their interest in an intervention that does not exist yet. Also, the majority of the participants had no experience with e-Health interventions. We have tried to overcome these problems by using mock-ups. The participants responded well to these mock-ups and they said that these were helpful during the interview. We would therefore recommend the use of mock-ups, prototypes, or demonstrations to other researchers that are willing to develop a Web-based intervention.

### Conclusions

We conclude that a Web-based intervention can be a valuable addition to existing support initiatives for partners of cancer patients. Furthermore, it is important that there is more awareness for the challenging situation partners of cancer patients are facing. This study yields important information about the content and form of a Web-based intervention for partners of cancer patients. In particular, flexibility and a positive approach seem to be the most important features. Also, information should be provided about the content and nature of an intervention in order to overcome misconceptions.
